# Cultural context in medical ethics: lessons from Japan

**DOI:** 10.1186/1747-5341-1-4

**Published:** 2006-04-03

**Authors:** Tia Powell

**Affiliations:** 1 New York State Task Force on Life & the Law, New York, NY, USA

## Abstract

This paper examines two topics in Japanese medical ethics: non-disclosure of medical information by Japanese physicians, and the history of human rights abuses by Japanese physicians during World War II. These contrasting issues show how culture shapes our view of ethically appropriate behavior in medicine. An understanding of cultural context reveals that certain practices, such as withholding diagnostic information from patients, may represent ethical behavior in that context. In contrast, nonconsensual human experimentation designed to harm the patient is inherently unethical irrespective of cultural context. Attempts to define moral consensus in bioethics, and to distinguish between acceptable and unacceptable variation across different cultural contexts, remain central challenges in articulating international, culturally sensitive norms in medical ethics.

## 

This paper examines two quite different topics in Japanese medical ethics: non-disclosure of medical information by Japanese physicians, and the history of human rights abuses by Japanese physicians during World War II. An analysis of these contrasting issues shows how culture shapes our view of ethically appropriate behavior in medicine. A grasp of cultural context reveals that certain practices, such as withholding diagnostic information from patients, may represent ethical behavior in that context. In contrast, nonconsensual human experimentation designed to harm the patient is inherently unethical irrespective of cultural context. The attempt to define moral consensus in bioethics, and to distinguish between acceptable and unacceptable variation across different cultural contexts, remain central challenges in articulating international, culturally sensitive norms in medical ethics.

## Non-disclosure of medical information

The non-disclosure of medical information by Japanese physicians is the most widely discussed topic in Japanese medical ethics, with more than 3000 articles published on this subject within a five year period [1]. Up until the 1980's, the unquestioned practice of Japanese doctors was to withhold a wide range of information from patients, particularly but not exclusively regarding cancer and poor prognoses. Diagnoses concealed from Japanese patients could extend to HIV seropositivity [2]. Psychiatric illness is highly stigmatized in Japan; for patients with schizophrenia, only 7% of psychiatrists in one study "always" informed a patient of the diagnosis [3]. When refering patients for psychiatric counseling, physicians might not reveal the nature of the referral, using instead vague terms such as "insomnia specialist " [4]. Reportedly, medicines of various types could be dispensed to patients with the labels removed [2].

Starting in the late 1980's and continuing today, Japanese physicians' practices regarding disclosure have been changing, with disclosure becoming more common, though not as prevalent as in the US. For instance, in a 1992 study of Japanese physicians, only 13% reported that they usually communicated cancer diagnoses to their patients [4]. More recent data show a marked and rapid increase in disclosure by Japanese physicians; one study documents an increase from 27% to 71% during the period from 1993 to 1998 [5].

Many factors contribute to the shift in disclosure practices in Japan. A widely reported medical malpractice case drew national attention to the potential for increased risk to patients because of nondisclosure. The case involved a nurse who was evaluated for abdominal pain in 1983 [4]. Her doctor diagnosed bladder cancer, but told the patient that she had gallstones and recommended surgery. The patient did not follow up with treatment for six months, by which time the cancer was no longer operable; the patient died several months later. A malpractice case ensued, unusual in Japan at that time [6]. The Japanese Supreme Court concurred with the physicians' choice to conceal the cancer diagnosis. The Court did urge physicians to select false diagnoses of equivalent urgency to true ones, so that patients will not unduly delay treatment [4].

Japanese society has devoted significant resources to the debate on disclosure practices. Two important official groups were convened and developed contrasting sets of recommendations in 1989 and 1990. The Government Task Force on Terminal Care was an appointed group including both physicians and non-physicians. It recommended that disclosure of cancer diagnoses be increased, though not without assessing four patient-related factors: psychological stability; decisional capacity; positive relationships between providers, family and patient; and available supports for the patient [7]. In contrast, the Bioethics Council of the Japan Medical Association, composed only of physicians, recommended against disclosure of cancer diagnoses, except in rare circumstances, i.e. when the patient excelled in all four factors listed above. Weakness in any single factor should preclude disclosure [7]. These two reports suggest that not only was Japan focused on the issue of disclosure, but that in the late 80's there was no general consensus on the ethically correct stance.

The Japanese government supports efforts to increase disclosure by way of a 1996 Ministry of Health regulation, revised in 1997, that permits physicians to bill the Japanese national health system specifically for documented disclosure of the patient's diagnosis and treatment plan [8]. In a nationalized system where physician fees are tightly controlled and are far lower than in the U.S., additional billing options provide a strong inducement toward disclosure.

The emotional tenor of discussions about disclosure in Japan is reflected by surgeon and hospice advocate Fumio Yamazaki's best-selling book, *Dying in a Japanese Hospital *[9]. Yamazaki gained notoriety as a disclosure advocate, arguing in his book that some patients, though by no means all, should be informed of their terminal diagnoses. Yamazaki painstakingly confronts the criticism that disclosure can be harmful to patients; he goes so far as to describe a case in which disclosure proved emotionally disastrous for both patient and physician. This case involved an elderly woman with extreme cancer-related pain, who demanded repeatedly and forcefully to be told her true diagnosis. Dr. Yamazaki did so, though without using the word cancer, in the presence of her daughter-in-law, who supported the patient with tearful embraces. Subsequently, the doctor describes the following events:

[The patient] didn't say a word to me or to the nurses for 24 hours after that. No matter how many times we spoke to her, she turned away and kept silent. Did she lose her speech because she was so shocked with knowing the truth? Or did she lose her speech because she was enraged with knowing how people she had trusted had betrayed her? [9]

When the patient's oldest son visited the hospital later that day, he attacked the doctor for breaking a promise to the family and telling his mother the truth. Dr. Yamazaki's pain in this encounter, and his sense of having tried *and failed *to act as an ethical physician by disclosing the patient's diagnosis is palpable.

The tone of the bioethics literature on nondisclosure in Japan can be harshly judgmental. Various articles attribute nondisclosure exclusively to the arrogance and paternalism of Japanese physicians [1,6,7]. Those who strive to increase disclosure portray themselves or are portrayed as reformers [7]. Repeatedly, comparisons are made between rates of disclosure in Japan and rates in the United States or elsewhere at an earlier date, with the implication that Japan lags behind the West by just this many years [2,7]. One account asserts that the Japanese practice of nondisclosure lacks any basis in principled behavior, but merely reflects culturally based conventions in which doctors have greater social status than many patients [1]. According to this view of nondisclosure, paternalism, hierarchy, and a lack of respect for patients provide a full explanation.

However, differences in social status between doctors and patients surely do not explain why Emperor Hirohito, who had been revered as a living god, fell ill from cancer in 1988, and did not have his diagnosis explicitly revealed to him [10]. It is widely assumed that the Emperor did eventually know his diagnosis, since he could observe the nature of his treatments and the reactions of others. The Emperor's physicians presumably relied upon *ishin denshin*, the Japanese term for non-verbal communication, which is viewed as appropriate and effective in many circumstances, particularly highly emotional ones [1,11]. Such indirect communication permits people to selectively avoid painful and unnecessary information and discussion.

Those unfamiliar with Japanese culture may see only the overt behavior, non-disclosure, and rush to the judgment that Japanese patients are treated disrespectfully by their physicians. However, knowledge of Japan's cultural context reveals that the ethical propriety of disclosure practices is more nuanced than is captured in some of the bioethics literature. For instance, when Japanese physicians practice nondisclosure, they generally do not conceal medical information from all concerned parties. Families, rather than patients, are often informed about diagnoses; decisions about whether or not to tell the patient are left to family members, who often decide against disclosure [2,4,7,12]. This process occurs irrespective of a patient's decision-making capacity. Michael Fetters argues that this practice is not an abrogation of autonomy, but a different form of autonomy, which he labels "family autonomy" [13]. In this paradigm, now the subject of societal debate and revision, disclosure to families is consistent with the assumptions and preferences of patients, families and physicians. Japanese physicians defer to family judgments about patients' coping skills and support systems. Only rarely would a Japanese physician inform a patient against the wishes of the family.

Is the idea of "family autonomy" a contradiction in terms? At first glance, an arrangement that precludes independent individual action seems inconsistent with autonomy. However, for individuals who prize group identity and welfare then a choice reached by consensus may be the best expression of the individual's values. From this perspective, we might say that individuals can express autonomy by delegating authority to the family.

Surveys of Japanese views on disclosure reveal interesting anomalies. In the 1990s, surveys reported that a large percentage of Japanese wanted to know their own diagnosis but would not want a family member informed. Fetters describes this as the "cancer disclosure paradox" [13]. He argues that the desire of individuals to know their own diagnosis suggests the increasing influence of individualism and a desire for personal autonomy. At the same time, notions of family responsibility support continued nondisclosure for significant others. In this view, a Japanese family cares for its members by shielding them from hurtful information. To permit a loved one to be informed without family assessment and approval is to fail in a crucial moral duty within Japanese and many cultures [14,15]. Though Japanese patients may wish disclosure for themselves, they believe they should protect family members from similar, potentially harmful direct disclosure.

Both taking care of others and allowing others to take care, including by making decisions, is accepted in a variety of Japanese cultural contexts. For instance, the concept of *omakase *describes the practice of placing oneself in the care of a professional [11]. In a measure of respect and trust, the client invites the professional to take responsibility for decision-making. The professional accepts this responsibility as a sign that the client depends on him, and is deserving of the utmost concern. This form of interaction exists within traditional Japanese doctor-patient relationships. It also exists in some Japanese restaurants, in which patrons acknowledge their respect for the chef by allowing him to choose for them. The chef's ability to know clients' wishes better even than they themselves know is a measure of the chef's skill. In fact, deference to professional wisdom is not limited to Japan; Americans when ill may also defer to the physician's judgment in a manner that resembles the concept of *omakase*.

Medical anthropologist Susan Long argues that the shift toward disclosure reflects the dissolution of a shared moral consensus among Japanese physicians, families and patients [16]. Current practices do not rise to the level of a shared view of morally correct actions regarding disclosure, but reveal both variability and uncertainty. This shift in disclosure practices represents an opportunity to view a society and its members in the midst of a "moral passage" away from one standard and toward a new one, as yet imperfectly defined [16].

Long addresses the interplay of disclosure and autonomy in offering a culturally sensitive analysis of Japanese practices [14]. She questions whether full disclosure is appropriate in the Japanese context, or merely an ill-suited Western imposition, and finds that both systems have much to learn from the other. Japanese physicians, patients and families are paying increasing attention to issues of patient autonomy. Americans, on the other hand, may learn by re-evaluating the emphasis on the doctor-patient dyad, which may undermine true autonomy by ignoring the importance of family participation in the lives of Americans. Other authors suggest that predominant Western models, contrasting autonomy and paternalism, fail to capture the range of options in the Japanese doctor-patient relationship [17].

A number of authors have criticized Western and particularly American bioethicists who place excessive value in individual autonomy [18]. Indeed, some bioethicists argue that U.S. practices inappropriately exclude families from a role in decision-making, while excessively focusing on individuals [19,20]. Such authors wish to borrow from other cultures a more inclusive approach to the role of families [18].

Patients who wish for disclosure and shared decision-making should have physicians who respect their wishes. However, the practice of limited medical disclosure remains common across most of the world today [15]. Nondisclosure cannot be attributed solely to physician paternalism, for this practice may also reflect the preferences of patients and families. Some patients do not wish for full disclosure; it is hard to argue that such patients *should *behave as Western bioethicists would like them to do, and that their physicians ought to impose autonomy-based relationships upon them. Some authors suggest that physicians need only inquire of patients about their desires for information, and then proceed accordingly. However, this practice stems from Western assumptions about the benefit of frank discussion. In a model where it is assumed that many important things will not and must not be spoken, the invitation to frank discussion may itself be an affront [21].

Japanese medical practices regarding disclosure are shifting. More and more Japanese patients and physicians expect and value candid discussion of medical information [5]. Yet does the shift toward disclosure prove that it is in some way *more *ethical than traditional practices? Perhaps this shift merely documents that Japanese practices are coalescing with Western ones, in a manner that does not prove the intrinsic worth of one practice versus another. Indeed, some bioethicists argue for an approach to disclosure that attempts to balance respect for cultural differences while encouraging shared decision-making when patients choose it [18]. It remains to be seen whether Japanese disclosure practices, once the current phase of rapid change resolves, end up looking exactly like American practices or like a new form.

In sum, full disclosure does not rise to the level of a universal norm. It was not the accepted standard in medicine for the past thousands of years, and is not currently the standard in most of the world. Nondisclosure is ethically appropriate for some patients, partly because of culturally shaped norms. Fuller disclosure is best for other patients because of their individual culturally shaped preferences.

A practice may be culturally accepted, yet still ethically unacceptable to others outside that culture. Practices like female circumcision arguably fall into this category. However, nondisclosure of medical information, when conforming to cultural expectations and benefiting the patient, need not be unethical. Other practices are not ethically permissible, and cannot be made so by any degree of cultural sensitivity. Egregious human rights abuses can not be understood as ethical regardless of context, as we shall see in the review of Japanese physician participation in biological warfare experiments during World War II.

## War crimes by Japanese physicians

Japanese physicians during World War II undertook biological warfare research that perpetrated significant abuse of human subjects. These acts cannot be understood as consistent with some different standard of medical ethics; they profoundly violated the standards of medical ethics even in that time and place. Indeed, the lead physician and researcher in Japan's biological warfare efforts, Shiro Ishii, addressed this dilemma in welcoming new staff members, acknowledging that their work was "completely opposite" to the physician's ethical obligation to cure disease [22]. A review of the abuse of human subjects in World War II confirms that cultural context cannot justify any and all behavior. Moreover, the abuse of research subjects is not limited to one culture. Abuse by Nazi physicians is widely known, and abuse in US government sponsored research is also well documented [23]. Bioethicists should study the cultural influences that promote such abuses in order to prevent recurrence.

Crimes by Japanese physicians were not publicized as were those by Nazi physicians, nor were Japanese physicians tried in the Tokyo War Crimes trials. For the most part, these acts have only gradually come to light in the decades after the war, due to the work of a handful of Japanese and other historians [24,25]. Significant additional evidence regarding Japanese wartime activities comes from the voluntary testimony of aging researchers and other staff who have stepped forward in the last decade, particularly in response to a traveling exhibit on biological warfare research, which toured 61 locations in Japan during 1993 and 1994 [26]. Japanese television documentaries have also publicized wartime biological warfare activities [22].

Japanese physicians and scientists undertook a massive program of biological warfare during WWII. A focus of this activity was a facility known as Unit 731, whose headquarters near Harbin, China, ultimately grew to encompass about 100 buildings, was under the direction of Shiro Ishii [27]. Research at Unit 731 took as its starting point a simple and correct observation: more soldiers die in wartime from disease than in battle. Ishii supervised defensive research that would prevent disease in Japanese soldiers, as well as research that would increase disease in civilian and military enemies of Japan. Substantial amounts of research at Unit 731 included medical experiments performed on prisoners, often causing terrible suffering. Subjects died either as a result of experiments or were put to death when no longer useful. Some prisoners were combatants, while others were captured civilians, including Chinese and Russian locals and Korean "comfort women." Witnesses also report seeing infants used as subjects in fatal experiments [26].

Research aimed at prevention of disease produced innovations at Unit 731, but at the cost of human life. Under Dr. Ishii's direction, researchers invented a portable water filtration system that allowed Japanese soldiers to protect their water supply while in contested areas or makeshift camps. Researchers studying frostbite gained knowledge about treatment by means of cruel experiments. They tied up prisoners in subzero temperature until severely frostbitten, then subjected the prisoners to various experiments, either to study potential treatments or to understand the physiology of freezing; prisoners died both due to frostbite and due to the experimental treatments [27]. Principle researchers in this work had illustrious post-war careers, with some serving on prestigious committees; one Unit 731 researcher served after the war on a prestigious committee on frostbite research, and ultimately became president of a publicly funded medical school [26,27].

Research was conducted on sexually transmitted disease, always the scourge of armies. Unit 731 staff members reported witnessing forced sex acts by infected prisoners, including Korean comfort women. Former staff of Unit 731 have testified that vivisection was a common means of studying disease processes [26].

Unit 731 conducted substantial research into biological warfare, a violation of existing treaty obligations. Such research focused on methods of disseminating disease agents such as cholera, bubonic plague, and malaria. Indeed, residents in the area of Harbin, China, reported outbreaks of bubonic plague for decades after the war; these outbreaks were the subject of a lawsuit of Chinese citizens against the Japanese government [28]. Unit 731 researchers designed various bombs for biological warfare; these bombs were used in China and against Soviet troops [23]. Bombs carrying infected fleas were dropped in areas where prisoners were tied to stakes, and careful measurements were taken in order to determine the efficacy of different methods. Civilian water supplies in local areas were also reportedly subject to intentional infection as part of biological warfare research [26]. Large numbers of research subjects as well as local residents are said to have died as a result of intentional exposure to toxic agents, either as a way to study the natural history of a disease, or its most effective route of transmission [22].

Japanese physicians do not appear to have planned a wholesale program of genocide as did the Nazis. Nonetheless, war crimes in the form of the horrific abuse of human subjects did occur and were known at the end of the war. Why then were these crimes, similar to those of the Nazi physicians, not publicized by trials like those at Nuremberg? A number of factors contributed to protect the secrecy of these crimes and their perpetrators. As the war neared its end, Unit 731 was dismantled with orders to preserve secrecy; there were no survivors among those incarcerated at Unit 731. Research records were destroyed, remaining prisoners were executed, and bodies were burned to destroy evidence.

However, not all evidence of Unit 731 was successfully suppressed. A few photographs remain, including photographs of bodies stacked and awaiting incineration. Likewise, some documents, including hundreds of autopsy reports of murdered research subjects, survived the attempt to destroy evidence [22]. Furthermore, numerous staff members were apprehended and interrogated; the Soviets held trials of staff from Unit 731 [24]. Some Japanese physicians and others served prison terms for as long as 11 years in China and the Soviet Union [27,23].

Most of the senior staff of Unit 731, including Dr. Ishii, returned to Japan as the war ended. These highly ranked staff members came under the exclusive jurisdiction of US military forces at the end of the war. America, already at the start of the Cold War, was reluctant to share secrets of biological warfare with its erstwhile allies, the Soviets and the Chinese. American military authorities chose not to include Dr. Ishii or any other participant in medical atrocities in the public war crimes trials in Tokyo, apparently in the hope of acquiring and maintaining the secrecy of valuable information from research into biological warfare [27]. Such a policy was consistent with lenient treatment arranged for various German scientists after the war [23]. However, current scholarship casts doubt on the value of information obtained from Dr. Ishii, and suggests rather that US military authorities bungled both the process of debriefing him and the option of trying him for war crimes [23]. Dr. Ishii negotiated clemency for himself and a range of senior workers, and lived out the post-war years at his home in Tokyo, ultimately dying of cancer.

Tanaka and other authors carefully weigh the issue of why Japanese physicians were willing to grossly abuse human subjects in the pursuit of research [27]. Drawing upon the concept of "doubling" used by Robert Jay Lifton in analyzing the motives of Nazi physicians [29], Tanaka argues that Japanese physicians were able to ignore the suffering of captive research subjects by creating a kind of separate self who conducted research, different from the self in other contexts. The researchers could follow different ethical rules from those of other people, since they were working to support the Emperor and Japan. All other ethical norms, including the norms of medical ethics, were subordinate to these goals. Research subjects were viewed not as suffering humans, but as non-human experimental material. Infamously, researchers at Unit 731 referred to their prisoners as "maruta," the Japanese word for log. Neither medical training nor medical ethics prevented these doctors from succumbing to the psychological and military structures that constitute "atrocity-producing situations" [30].

## Conclusion

This paper examines two contrasting issues in Japanese medical ethics. The first section analyzes the workaday issue of how physicians share information with patients, and argues that a range of practices may suit ethical norms in different cultural contexts. The second topic, the gross abuse of human subjects during wartime, is one that fewer physicians will directly confront. However, the collaboration of physicians in the abuse of prisoners of war does recur.

Non-disclosure of medical information to patients can constitute an ethical practice when certain conditions apply. First, the overt and credible intent of the physician in withholding information must be to benefit the patient. Withholding diagnostic information to protect a third party, for instance to conceal medical error, does not meet criteria for ethical behavior. Second, the patient must also view this practice as beneficial. Ascertaining the patient's view is inherently difficult when medical practices cannot be frankly discussed. However, either through the patient's expressed views or the family's interpretation of those views, patients in the past and to a lesser extent today indicate that they view the withholding of certain types of medical information as beneficial. Third, the patient must at least tacitly agree to the practice of withholding information in the current instance. In a culture where such practices are the norm, physicians may have greater confidence in family statements about the patient's beliefs. However, members of a culture may accept a practice as applied to others but not to themselves. In the current climate of change, careful assessment of beliefs with both patients and families provide an ethical foundation for selecting the best approach to disclosure.

By contrast, the abuse of research subjects as documented in Unit 731 can never be ethical. Research, unlike medical treatment, poses an inherent ethical challenge, in that it is never only in the patient's interest, but always brings with it potentially competing claims, such as the good of other patients in the future. Consent is important for ethical treatment, yet even more critical in the ethical practice of research, as noted in various codes of research ethics. In the case of Unit 731, no pretense of consent was made; the subjects were held as prisoners without rights, whether they were civilians or combatants, adults or children. Neither was there any pretense of benefit to the subject; the overt goal was to use the lives of these subjects in order to gain benefit for others. No recorded attempt was made to preserve the life or minimize the suffering of the victims. It is possible, though far from certain, that a cultural standard in Japan during WWII may have supported such abuse of prisoners. Japanese citizens and soldiers made enormous sacrifices to support the war, even to the point of committing suicide. However, it is not enough for a standard to exist in a culture for it to serve as the basis of an ethical practice; it must at a minimum be accepted by those to whom it is applied in the specific instance. Since no prisoner of Unit 731 was there voluntarily, none can be said to have accepted the role of subhuman research material.

Contemporary emphasis on cultural sensitivity and cultural competence enriches bioethics by broadening the understanding of ethical practices internationally. A greater awareness of practices around the world leads to modesty in asserting the ethical universality of certain practices, such as modes of communicating medical information. Many aspects of medical practice are deeply shaped by culture; the meaning of illness, the nature of healing, and styles of communication between doctor, patient, and family are all bound by culture and language. Yet it is important to distinguish between cultural competency and cultural relativism. Understanding the context of a time and place may permit us to discern ethical principles behind behaviors that at first strike us as merely wrong. However, other behaviors will remain ethically unacceptable regardless of our grasp of the cultural context. Indeed, the rational for some behaviors may elude understanding, irrespective of efforts to place motives within a context. Behavior during wartime, either by physicians or others, can fall to a level of barbarism that defies acceptance or understanding. The tragedy is that the capacity for egregious behavior under extraordinary circumstances exists in a wide range of cultures and peoples, our own included.

## References

[B1] Konishi E, Davis A (1999). Japanese nurses' perceptions about disclosure of information at the patients' end of life. Nursing and Health Sciences.

[B2] Mitsuya H (1997). Telling the truth to cancer patients and patients with HIV-1 infection in Japan. Annals of the New York Academy of Sciences.

[B3] Ono Y, Satsumi Y, Kim Y, Iwadate T, Moriyama K, Nakane Y, Nakata T, Okagami K, Sakai T, Sato M, Someya T, Takagi S, Ushijima S, Yamauchi K, Yoshimura K (1999). Schizophrenia: Is it time to replace the term?. Psychiatry and Clinical Neurosciences.

[B4] Uchitomi Y, Yamawaki S (1997). Truth-telling practice in cancer care in Japan. "Communication with the cancer patient: Information and truth" Annals of the New York Academy of Sciences.

[B5] Horikawa N, Yamzaki T, Sagawa M, Nagata T (2000). Changes in disclosure of information to cancer patients in a general hospital in Japan. General Hospital Psychiatry.

[B6] Morikawa I (1994). Patients' rights in Japan: progress and resistance. Kennedy Institute of Ethics Journal.

[B7] Tanida N (1994). Japanese attitudes towards truth disclosure in cancer. Scandinavian Journal of Social Medicine.

[B8] Akabayashi A, Fetters M (2000). Paying for informed consent. Journal of Medical Ethics.

[B9] Yamazaki F (1996). Dying in a Japanese Hospital.

[B10] Hattori H (1991). Patients' rights to information. Social Science and Medicine.

[B11] Suzuki S, Kirschling J, Inoue I (1993). Hospice Care in Japan. American Journal of Hospice & Palliative Care.

[B12] Hamajima N, Tajima K, Morishita M, Hyodo C, Sakakibara N, Kawai C, Moritaka S (1996). Patients' expectations of information provided at cancer hospitals in Japan. Japanese Journal of Clinical Oncology.

[B13] Fetters M (1998). The family in medical decision-making: Japanese perspectives. Journal of Clinical Ethics.

[B14] Long S (1999). Family surrogacy and cancer disclosure: physician-family negotiation of an ethical dilemma in Japan. Journal of Palliative Care.

[B15] Kagawa-Singer M, Blackhall L (2001). Negotiating cross-cultural issues at the end of life: You gotta go where he lives. Journal of the American Medical Association.

[B16] Long S (2000). Public Passages, Personal Passages, and Reluctant Passages: Notes on Investigating Cancer Disclosure Practices in Japan. Journal of Medical Humanities.

[B17] Elwyn T, Fetters M, Gorenflo D, Tsuda T (1998). Cancer Disclosure in Japan: Historical Comparisons, Current Practices. Social Science and Medicine.

[B18] Blackhall L, Murphy S, Frank G, Michel V, Azen S (1995). Ethnicity and Attitudes toward patient autonomy. Journal of the American Medical Association.

[B19] Nelson HL, Nelson JL (1995). The Patient in the Family.

[B20] Ruhnke G, Wilson S, Akamatsu T, Kinoue T, Takashima Y, Goldstein MK, Koenig B, Hornberger J, Raffin T (2000). Ethical Decision Making and Patient Autonomy. Chest.

[B21] Elwyn T, Fetters M, Sasaki H (2002). Responsibility and Cancer Disclosure in Japan. Social Science and Medicine.

[B22] Harris S (2002). Factories of Death.

[B23] Moreno J (2001). Undue Risk: Secret State Experiments on Humans.

[B24] Dower J (1999). Embracing Defeat.

[B25] Harris S (1994). Factories of Death: Japanese Biological Warfare, 1932–45, and the American Cover-up.

[B26] Gold H (1996). Unit 731 Testimony.

[B27] Tanaka Y (1996). Hidden Horrors.

[B28] Watts J (2002). Court forces Japan to admit to dark past of bioweapons programme. Lancet.

[B29] Lifton RJ (1986). Nazi Doctors: Medical Killing and the Psychology of Genocide.

[B30] Lifton RJ (2004). Doctors and Torture. New England Journal of Medicine.

